# *Parkia speciosa* Hassk. Empty Pod Extract Prevents Cardiomyocyte Hypertrophy by Inhibiting MAPK and Calcineurin-NFATC3 Signaling Pathways

**DOI:** 10.3390/life13010043

**Published:** 2022-12-23

**Authors:** Nor Hidayah Mustafa, Juriyati Jalil, Mohammed S. M. Saleh, Satirah Zainalabidin, Ahmad Yusof Asmadi, Yusof Kamisah

**Affiliations:** 1 Centre for Drug and Herbal Development, Faculty of Pharmacy, Universiti Kebangsaan Malaysia, Kuala Lumpur 50300, Malaysia; 2 Department of Pharmacology, Faculty of Medicine, Universiti Kebangsaan Malaysia, Cheras, Kuala Lumpur 56000, Malaysia; 3 Program of Biomedical Science, Centre of Applied and Health Sciences, Faculty of Health Sciences, Universiti Kebangsaan Malaysia, Kuala Lumpur 50300, Malaysia; 4 Unit of Pharmacology, Faculty of Medicine and Defence Health, Universiti Pertahanan Nasional Malaysia, Kem Sungai Besi, Kuala Lumpur 57000, Malaysia

**Keywords:** *Parkia speciosa*, cardiac hypertrophy, angiotensin II, cardiac remodeling

## Abstract

Cardiac hypertrophy is an early hallmark during the clinical course of heart failure. Therapeutic strategies aiming to alleviate cardiac hypertrophy via the mitogen-activated protein kinase (MAPK)/calcineurin-nuclear factor of activated T-cells (NFAT) signaling pathway may help prevent cardiac dysfunction. Previously, empty pod ethanol crude extract of *Parkia speciosa* Hassk was shown to demonstrate protective effects against cardiomyocyte hypertrophy. Therefore, the current study aimed to investigate the effects of various fractions of the plant ethanol extract on the MAPK/NFAT signaling pathway in angiotensin II (Ang II)-induced cardiomyocyte hypertrophy. Simultaneous treatment with ethyl acetate (EA) fraction produced the most potent antihypertrophic effect evidenced by the reduced release of B-type natriuretic peptide (BNP). Subsequently, treatment with the EA fraction (6.25, 12.5, and 25 μg/mL) prevented an Ang II-induced increase in cell surface area, hypertrophic factors (atrial natriuretic peptide and BNP), reactive oxygen species, protein content, and NADPH oxidase 4 expression in the cells. Furthermore, EA treatment attenuated the activation of the MAPK pathway and calcineurin-related pathway (GATA-binding protein 4 and NFATC3), which was similar to the effects of valsartan (positive control). Our findings indicate that the EA fraction prevents Ang II-induced cardiac hypertrophy by regulating the MAPK/calcineurin-NFAT signaling pathway.

## 1. Introduction

Heart failure has become a major global clinical problem that claimed approximately 17.9 million lives in 2019 [1]. Causes of heart failure, including long-standing hypertension and coronary artery disease, may arise from unhealthy dietary habits and a sedentary lifestyle [2]. Pathological cardiac hypertrophy is a prerequisite marker of heart failure. It is characterized by the thickening of cardiac muscle, leading to irreversible morphological and functional changes. Following the induction of the hypertrophic response, fetal cardiac genes—atrial natriuretic peptide (ANP), B-type natriuretic peptide (BNP), and β-myosin heavy chain—are activated. They are well-known cardiac hypertrophy biomarkers that increase with the progression of cardiac hypertrophy, ultimately causing heart failure [3]. Numerous mediators and signaling pathways are involved in the development of cardiac hypertrophy. The mitogen-activated protein kinase (MAPK) cascade comprises three major subfamilies: p38 kinase, c-Jun N-terminal protein kinase (JNK), and extracellular signal-regulated protein kinase (ERK1/2). The cascade produces reactive oxygen species (ROS) via NADPH oxidase (Nox) in the cardiovascular system; Nox plays a significant role in the pathogenesis of cardiac hypertrophy, with increased expression of Nox2 and Nox4 in cardiomyocytes [4,5]. Direct inhibition of the MAPK pathway significantly decreases cardiac hypertrophy [6,7,8]. Accumulating data demonstrate that the activation of the calcium-calcineurin pathway and its downstream target—nuclear factor of activated T cells (NFAT)—are well-established as central prohypertrophic factors in the regulation of myocardial hypertrophy [9]. Upon stimulation of the hypertrophic response, calcineurin dephosphorylates NFAT transcription factor, which then translocates into the nucleus to form a complex with GATA-binding protein 4 (GATA4), a key molecule modulating cardiac hypertrophy-related genes. The MAPK and calcium-calcineurin pathways serve as a key regulatory axis in cardiac hypertrophy, and they are involved in an interdependent network of signaling cascades [10,11,12].

Heart failure is clinically treated using various drugs, such as angiotensin receptor blockers, β-blockers, and angiotensin receptor-neprilysin inhibitors [13]. Extensive studies have been performed on medicinal plants, investigating their potential in reducing cardiac hypertrophy as an alternative to modern medicines. Extracts from *Parkia speciosa* Hassk. empty pods [14], *Plantago asiatica* L. seeds [15], and *Centella asiatica* (L.) Urb. [16] were demonstrated to decrease cardiac hypertrophy.

*P. speciosa* Hassk. (Fabaceae), also known as the bitter bean, is a perennial tree that grows abundantly in Southeast Asia. It is traditionally used to alleviate hypertension and maintain cardiovascular health [17]. The plant’s empty pod possesses diverse biological properties, including antioxidant [18], anti-inflammatory [19,20], hypotensive [21], and antidiabetic [22,23] properties. These properties have been associated with the presence of several compounds, including phenolic acids, tannins, flavonoids (e.g., gallic acid, ellagic acid, quercetin, rutin, gossypetin, and catechin), triterpenes, and polysaccharides [23,24]. The crude ethanol extract of this plant exhibits a protective effect against cardiomyocyte hypertrophy by modulating the MAPK signaling pathway and the angiotensin II (Ang II)/ROS/nitric oxide (Ang II/ROS/NO) axis [14]. However, the effects of its fractions on other signaling pathways involved in the pathogenesis of cardiac hypertrophy, such as the calcineurin-NFAT signaling pathway, remain unclear. Therefore, the present study explored the co-treatment effects of various fractions of *P. speciosa* empty pod ethanol extract on the regulation of the MAPK and calcineurin-NFAT signaling pathways in Ang II-induced cardiomyocyte hypertrophy.

## 2. Materials and Methods

### 2.1. Plant Collection and Sequential Extraction of Empty Pods

*P. speciosa* pods were collected from Kampung Kua, Ajil, Terengganu, Malaysia (5°00′01.8″ N 103°04′24.6″ E) in February 2020. The plant was authenticated by a botanist and deposited at the Universiti Kebangsaan Malaysia Herbarium (Voucher specimen: ID009/2020). Extraction of the pods was performed following the methods outlined by a previous study [23]. Briefly, they were peeled, deseeded, cut into small pieces, and freeze-dried for five days. The dried, empty pods were crushed into powder and extracted in ethanol in a 1:10 (w/v) ratio before being sonicated (320 W, 30 °C) for 1 h. The solution was then centrifuged (5 min, 10,000 rpm), filtered with Whatman filter paper, and the filtrate was vacuum-evaporated at 40 °C. A part of the crude extract was further fractionated with *n*-hexane, chloroform, ethyl acetate, and methanol according to polarity. The extracts were dried with a rotary vacuum evaporator and freeze-dryer before being stored at −80 °C until further use.

### 2.2. Cell Culture

The H9c2 cell line from rat embryonic myocardial ventricles was acquired from the American Type Culture Collection (ATCC CRL-1446, Rockville, MD, USA). The cells (passages 3–7) were cultured in high-glucose Dulbecco’s modified Eagle medium (DMEM) (Gibco, Grand Island, NY, USA) complemented with fetal bovine serum (10%, Gibco, Grand Island, NY, USA) and penicillin/streptomycin (1%, 100 IU/mL:100 µg/mL) in humidified air with 5% CO_2_ (37 °C). The cells were starved in serum-free DMEM for 24 h before treatment.

### 2.3. Cell Viability Measurement

Cell viability was estimated using 3-(4,5-dimethylthiazol-2-yl)-5-(3-carboxy-methoxyphenyl)-2-(4-sulphenyl)-2*H*-tetrazolium assay (Promega, Madison, WI, USA). H9c2 cells (1 × 10^5^ cells/dish) were exposed to various concentrations of crude ethanol extract and *n*-hexane, chloroform, ethyl acetate, and methanol fractions for 24 h. The fractions were liquefied in dimethyl sulfoxide (DMSO) (Sigma-Aldrich, St. Louis, MO, USA), with a final culture concentration of the solvent being less than 0.001%.

### 2.4. Antihypertrophic Screening of Extract Fractions

The potential antihypertrophic effect of the crude extract and various *P. speciosa* empty pod fractions was screened in H9c2 cardiomyocytes that were co-incubated with 1 µM Ang II [25] for 24 h. The concentrations of the extract and fractions we used were lower than their respective IC_50_ values. BNP level was determined using a commercial assay kit (Elabscience, Houston, TX, USA) to assess cell hypertrophy, calculated against a standard curve. The fraction that exhibited the highest antihypertrophic property was selected for subsequent experiments to elucidate its cardioprotective mechanisms.

### 2.5. Chromatography Analysis

Phytochemical profiling of the ethyl acetate fraction was performed using highly sensitive ultra-high-performance liquid chromatography-quadrupole time of flight-mass spectrometry (UHPLC-QTOF-MS) following a previously described method [23]. Chromatography analysis was performed using an Agilent 1290 Infinity II UPLC System (Agilent Technologies Santa Clara, CA, USA) and 6550 iFunnel Q-TOF LC/MS system (Agilent Technologies, Santa Clara, CA, USA) equipped with a C18 column (100 mm × 2.1 mm I.D.; 3-µm particle size) (Agilent Technologies, Santa Clara, CA, USA). Samples were eluted with 0.1% aqueous formic acid solution and methanol at 0.4 mL/min. The negative ion mode was selected due to its high selectivity and sensitivity for further liquid chromatography-mass spectrometry (LC-MS) analysis.

### 2.6. Experimental Groups

H9c2 cells (1 × 10^5^ cells/dish) were randomly divided into seven groups. Five groups were exposed to 1 µM Ang II (Sigma Aldrich, St Louis, MO, USA) [25]; three of these groups were co-treated with different concentrations of ethyl acetate fraction (6.25, 12.5, and 25 µg/mL) from the empty pod extract, and another group was co-treated with valsartan (20 μM) [26] as the positive control. The fraction and valsartan were dissolved in 0.001% DMSO. A group treated with vehicle (0.001% DMSO) served as the control, and another group was incubated with 25 µg/mL ethyl acetate fraction of the extract. Incubation with the respective treatments was performed for 24 h.

### 2.7. Cell Size Measurement

Briefly, H9c2 cells were inoculated in six-well plates (1 × 10^4^ cells/well). At the end of treatment, the cells were rinsed with phosphate-buffered saline (PBS). The cells were affixed with paraformaldehyde (4%) before being dyed with 0.5% crystal violet in ethanol (2%) [27]. The cross-sectional surface area of a minimum of ten representative cells from three wells were averaged and recorded as one *n* value. The area was quantified using Image J software (U. S. National Institutes of Health, Bethesda, MD, USA) and expressed as a ratio of the control cells.

### 2.8. Protein Content Assay

Protein content was measured as described previously [27,28]. H9c2 cells were lysed with radioimmunoprecipitation assay (RIPA) buffer before their protein content was estimated using a commercial kit (BioRad Laboratories, Hercules, CA, USA) and expressed as mg protein per 10^5^ cells.

### 2.9. ROS Assay

ROS production was measured using a 2′7′-dichlorodihydrofluorescein diacetate (DCFH-DA) cellular ROS detection assay kit (ab113851, Abcam, Cambridge, UK). H9c2 cells from each group were incubated with 20 µM DCFH-DA for 30 min at 37 °C in the dark. The cells were rinsed thrice before visualization under a fluorescence microscope (Olympus Optical, Tokyo, Japan) at 100× magnification. The cell intensity from six arbitrarily chosen areas was evaluated using ImageJ software (US National Institutes of Health, Bethesda, MD, USA).

### 2.10. NADPH Oxidase 4 (Nox4) Immunofluorescence Assay

To determine Nox4 expression, 24 h after treatment, the cells were affixed with paraformaldehyde (4%) in PBS at room temperature for 15 min before being permeabilized with 0.1% Triton X-100/0.1% sodium citrate for 20 min. The cells were blocked with bovine serum albumin (5%) and incubated with Nox4 antibody (1:200; sc-518092, Santa Cruz Biotechnology, Dallas, TX, United States) diluted in 1% skimmed milk solution overnight. Then, the cells were incubated with Alexa Fluor 488 goat anti-mouse antibody (1:200; A-11059, Invitrogen, Waltham, MA, USA) for 1 h and rinsed with PBS. The cellular nuclei were stained blue with 4′,6-diamidino-2-phenylindole (DAPI; ab228549, Abcam, Cambridge, MA, USA) and analyzed under a fluorescence microscope (Olympus Optical, Tokyo, Japan).

### 2.11. ANP and NFATC3 Gene Expression Assay

TRIzol reagent (Sigma-Aldrich, St. Louis, MO, USA) was used to extract total RNA from H9c2 cells 24 h after treatment. Then, 1 µg of total RNA was reverse-transcribed using ReverTra AceTM qPCR RTMaster Mix with gDNA Remover (FSQ-301; Toyobo, Japan) to obtain complementary DNA. RNA levels of ANP and NFATC3 in H9c2 cells were quantified using SYBR Green PCR Master Mix Kit (QPS-201; Toyobo, Japan). Glyceraldehyde-3-phosphate dehydrogenase (GAPDH) was employed as an in-house control. A quantitative reverse transcription polymerase chain reaction (RT-qPCR) was conducted using a CFX96TM Real-Time System (Bio-Rad Laboratories, CA, USA) for 60 cycles. The sequences of primers for amplification were: ANP, 5′-GGTAGGATTGACAGGATTGGA-3′ and 5′-GCAGATTTGGCTGTTATCTTCG-3′; NFATC3, 5′-GGAAATCCCACTTCTACCTGAA-3′ and 5′-GCCAATATCAGTTTCTCCTTTTCG-3′, and GAPDH, 5′-AACCCATCACCATCTTCCAG-3′ and 5′-CCAGTAGACTCCACGACATAC-3′. Cycle threshold (Ct) values were used to measure the amount of amplified PCR product relative to GAPDH as the control.

### 2.12. Protein Expression

The expression of proteins of interest was analyzed via western blotting. After treatment, cells were lysed in RIPA buffer (Sigma-Aldrich, St. Louis, MO, USA) containing a protease inhibitor cocktail (Roche, Basel, Switzerland) and phosphatase inhibitor (Roche, Basel, Switzerland). The protein content in supernatants obtained after 30 min of centrifugation at 14,000 rpm (4 °C) was quantified by using a Bradford assay (Sigma Aldrich, St. Louis, MO, USA). Proteins (30 μg) were applied to sodium dodecyl sulfate-polyacrylamide gel electrophoresis (10–12%) and then moved to polyvinylidene fluoride (PVDF) membranes (Bio-Rad Laboratories, Hercules, CA, USA). Membrane blocking was performed for 1 h using 5% w/v bovine serum albumin or 5% skimmed milk powder in 1% Tris-buffered saline/1% Tween 20. Subsequently, the PVDF membranes were incubated overnight at 4 °C with primary antibodies against phosphorylated p38 (p-p38, 1:1000; sc-166182, Santa Cruz Biotechnology, Dallas, TX, USA), phosphorylated ERK (p-ERK, 1:1000; #4377; Cell Signaling Technology, Danvers, MA, USA), phosphorylated JNK (p-JNK, 1:500; ab4821, Abcam, Cambridge, UK), phosphorylated GATA4 (p-GATA4, 1:1000; sc-32823, Santa Cruz Biotechnology, Dallas, TX, USA), calcineurin (1:1000; ab71149, Abcam, Cambridge, UK), Nox4 (1:1000; sc-518092, Santa Cruz Biotechnology, Dallas, TX, USA), and β-actin (1:1000; sc-47778, Santa Cruz Biotechnology, Dallas, TX, USA). After meticulous washing, the membranes were incubated with horseradish peroxidase-conjugated IgG secondary antibody (1:3000; anti-mouse 7076P2; anti-rabbit 7074S; Cell Signaling Technology, Danvers, MA, USA) for 1 h at room temperature. The bands were visualized via enhanced chemiluminescence developing solution (Bio-Rad, Hercules, CA, USA) using a gel documentation system (AI600RGB, GE Healthcare Japan Corporation, Tokyo, Japan). The bands were then quantified using ImageJ software (US National Institutes of Health, Bethesda, MD, USA).

### 2.13. Statistical Analysis

The Shapiro–Wilk test was used to assess the data distribution. The data were analyzed using one-way analysis of variance and Tukey’s post-hoc test using SPSS v24.0 software (IBM Corp., Armonk, NY, USA). Data are presented as mean ± standard error of the mean (SEM) from a minimum three independent triplicate assays. The accepted statistically significant value was *p* < 0.05.

## 3. Results

### 3.1. Cell Cytotoxicity and Antihypertrophic Screening of Fractions

**Figure 1:**
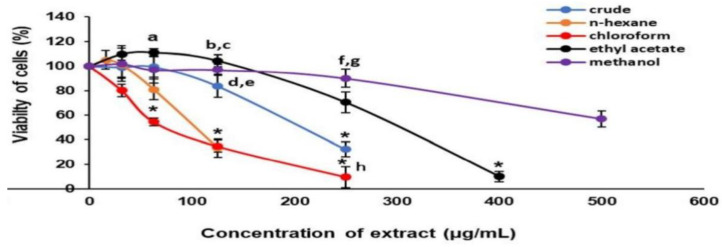
Effect of *Parkia speciosa* empty pod crude extract and fractions on H9c2 cardiomyocyte viability. The data are expressed as means ± SEM (*n* = 3). * *p* < 0.05 compared to control (0 µg/mL), ^a^ *p* < 0.05 compared to chloroform 62.5 µg/mL, ^b^ *p* < 0.05 compared to chloroform 125 µg/mL, ^c^ *p* < 0.05 compared to *n*-hexane 125 µg/mL, ^d^ *p* < 0.05 compared to chloroform 125 µg/mL, ^e^ *p* < 0.05 compared to *n*-hexane 125 µg/mL, ^f^ *p* < 0.05 compared to chloroform 250 µg/mL, ^g^ *p* < 0.05 compared to crude 250 µg/mL, ^h^ *p* < 0.05 compared to chloroform 250 µg/mL.

**Figure 2:**
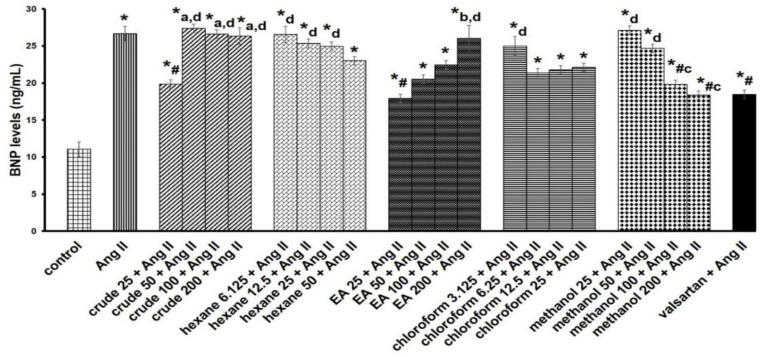
Effects of various concentrations (µg/mL) of *P. speciosa* empty pod crude extract and fractions, and valsartan (20 μM) on brain natriuretic peptide (BNP) in angiotensin II (Ang II)-induced cardiac hypertrophy in H9c2 cardiomyocytes. The data are expressed as means ± SEM. * *p* < 0.05 compared to control, ^#^ *p* < 0.05 compared to Ang II. EA, ethyl acetate. ^a^ *p* < 0.05 compared to crude 25 + Ang II, ^b^ *p* < 0.05 compared to EA 25 + Ang II, ^c^ *p* < 0.05 compared to methanol 25 + Ang II, ^d^ *p* < 0.05 compared to valsartan + Ang II.

The median inhibitory concentration (IC_50_) of the crude extract, *n*-hexane, chloroform, ethyl acetate, and methanol fractions was approximately 216.69 ± 5.66 µg/mL, 114.88 ± 7.46 µg/mL, 62.58 ± 3.29 µg/mL, 315.88 ± 4.92 µg/mL, and 531.92 ± 6.36 µg/mL, respectively (Figure 1). Preliminary antihypertrophic screening of extracts with cellular BNP levels demonstrated significantly increased antihypertrophic activity with crude (25 µg/mL), ethyl acetate (25 µg/mL), and methanol (100 and 200 µg/mL) fractions compared with the Ang II group (Figure 2). Based on these findings, an ethyl acetate fraction at 25 µg/mL was selected for subsequent experiments.

### 3.2. UHPLC-QTOF-MS/MS Identification

**Figure 3:**
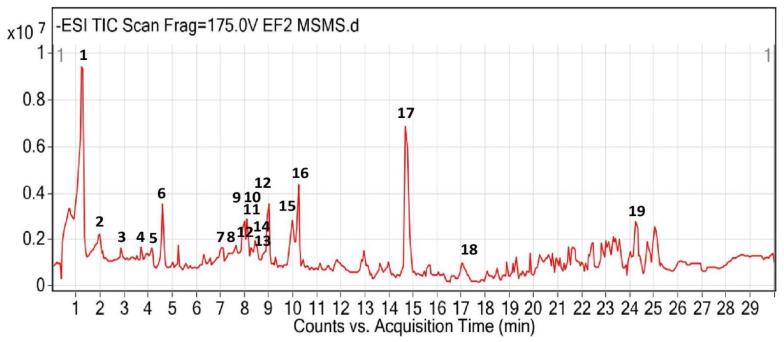
Total ion chromatogram of the ethyl acetate (EA) fraction of *P. speciosa* empty pods. **1**, Gallic acid; **2**, pantothenic acid; **3**, 2′-2′-bisepigallocatechin monogallate; **4**, theasinensin A; **5**, picein; **6**, thonningianin A; **7**, 3,5,7,2′,6′-pentahydroxyflavone 2′-glucoside; **8**, amaronol A; **9**, quercetin 3′-glucoside; **10**, quercetin 3-(6‴-sinapylglucosyl)(1->2)-galactoside; **11**, spiraeoside; **12**, kaempferol 3-alpha-D-galactoside; **13**, 6-hydroxyluteolin 7-apioside; **14**, gossypetin; **15**, herbacetin; **16**, bracteatin; **17**, 3′-N-debenzoyl-2′-deoxytaxol; **18**, glepidotin C; and **19**, mayolene-16.

The total ion chromatogram profile of the ethyl acetate fraction of *P. speciosa* empty pods is depicted in Figure 3. Based on the MS/MS database library, 19 compounds were characterized and predicted from the ethyl acetate fraction, including gallic acid, picein, amaronol A, and quercetin (Table 1). Most of the compounds were flavonoids.

### 3.3. Ethyl Acetate Fraction Inhibited Ang-II Induced Cardiomyocyte Hypertrophy

**Figure 4:**
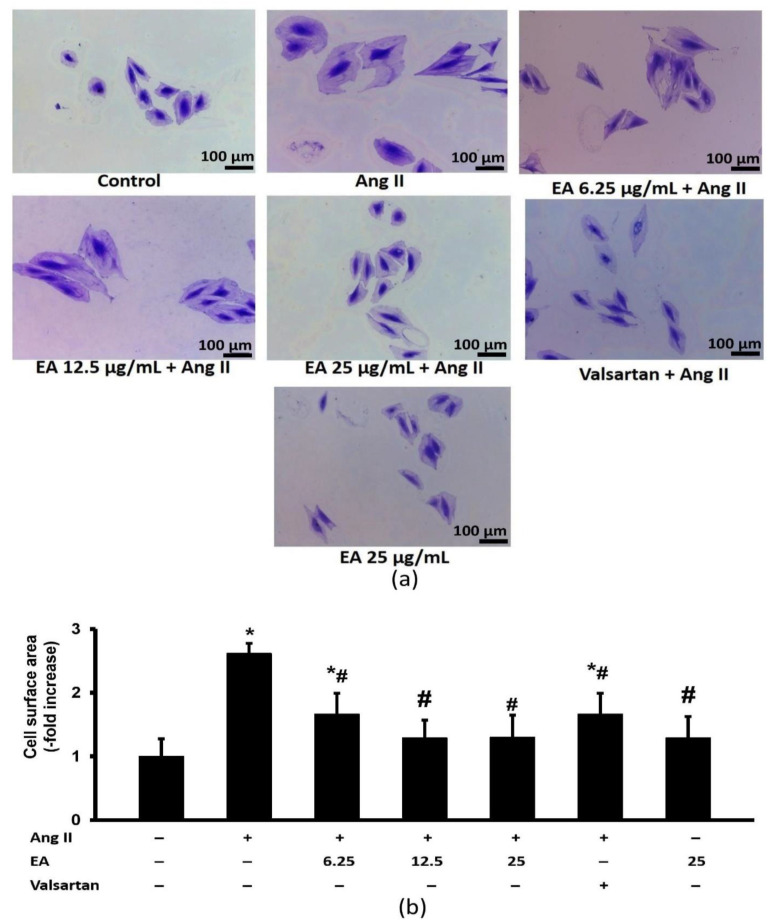
Effects of the EA fraction (µg/mL) of *P. speciosa* and valsartan (20 µM) on cell surface area in Ang II-induced cardiomyocyte hypertrophy. (**a**) Representative images from each group at 100× magnification and (**b**) relative cell surface area. Data are expressed as means ± SEM (*n* = 3). * *p* < 0.05 compared to control, ^#^ *p* < 0.05 compared to Ang II.

Ang II significantly enhanced the expansion of cell surface area (Figure 4). The effect was significantly decreased following treatment with various concentrations of ethyl acetate fraction of *P. speciosa* and valsartan. No significant difference was noted among the treated groups.

### 3.4. Ethyl Acetate Fraction Inhibited Ang II-Induced Increased Total Protein Content and ANP Expression

**Figure 5:**
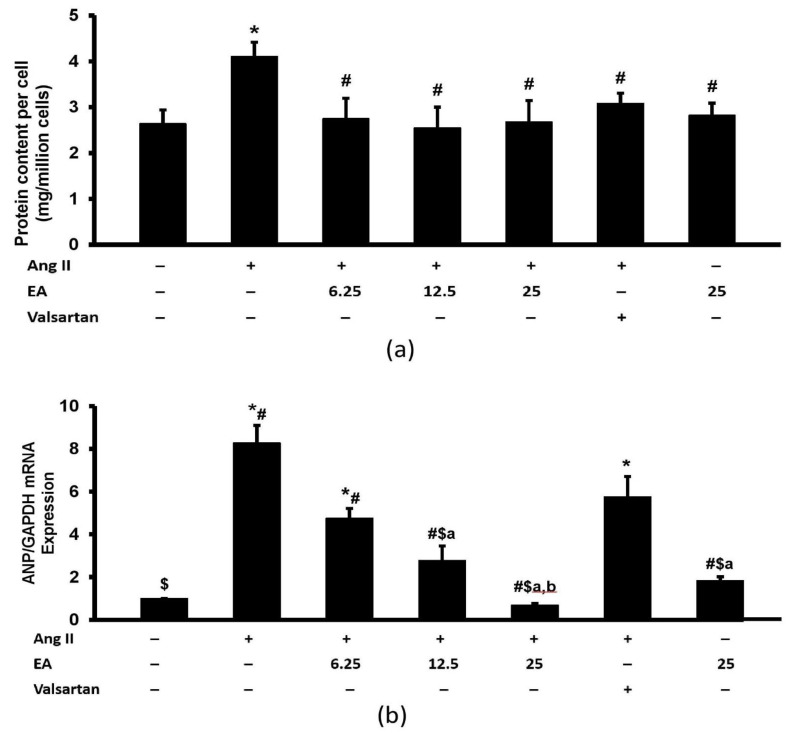
Effects of the EA fraction (µg/mL) of *P. speciosa* and valsartan (20 µM) on (**a**) protein content per cell (mg/million cells) and (**b**) ANP/GAPDH mRNA expression in Ang II-induced cardiomyocyte hypertrophy. Data are expressed as means ± SEM (*n* = 3). * *p* < 0.05 compared to control, ^#^ *p* < 0.05 compared to Ang II, ^$^ *p* < 0.05 compared to valsartan + Ang II, ^a^ *p* < 0.05 compared to 6.25 µg/mL EA + Ang II, ^b^ *p* < 0.05 compared to 12.5 µg/mL EA + Ang II.

Exposure to Ang II increased the total protein content (Figure 5a). The effect was significantly inhibited in the presence of ethyl acetate fraction and valsartan. No differences were noted among the treated groups. Furthermore, Ang II exposure increased the expression of fetal cardiac gene ANP in H9c2 cardiomyocytes (Figure 5b). Co-incubation with various concentrations of ethyl acetate fraction reduced this increase in a concentration-dependent manner. Moreover, valsartan exerted a significant protective effect on the parameter. The expression of ANP in the ethyl acetate fraction-treated groups, except for the lowest concentration (6.25 µg/mL), was significantly lower than in the valsartan group.

### 3.5. Ethyl Acetate Fraction Suppressed Ang II-Induced Intracellular ROS Generation

**Figure 6:**
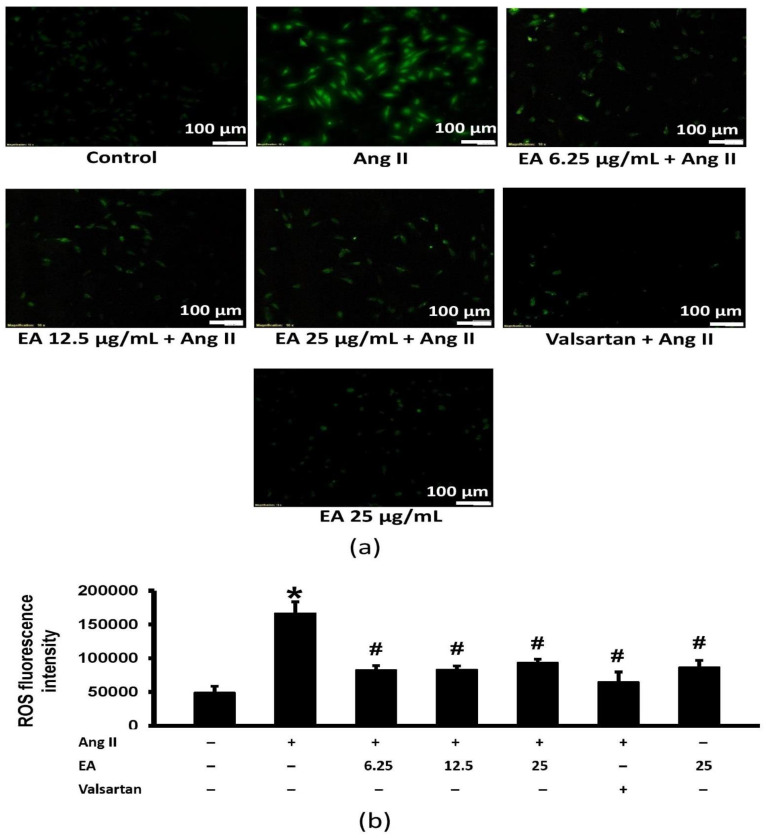
Effects of the EA fraction (µg/mL) of *P. speciosa* and valsartan (20 µM) on reactive oxygen species (ROS) in Ang II-induced cardiomyocyte hypertrophy. (**a**) Representative images from each treatment group and (**b**) ROS fluorescence intensity. Data are expressed as means ± SEM (*n* = 3). * *p* < 0.05 compared to control, ^#^ *p* < 0.05 compared to Ang II.

Ang II significantly increased intracellular ROS levels (Figure 6). Both the ethyl acetate fraction and valsartan reduced the Ang II-induced increase in ROS generation.

### 3.6. Ethyl Acetate Fraction Suppressed Ang II-Induced Nox4 Expression

**Figure 7:**
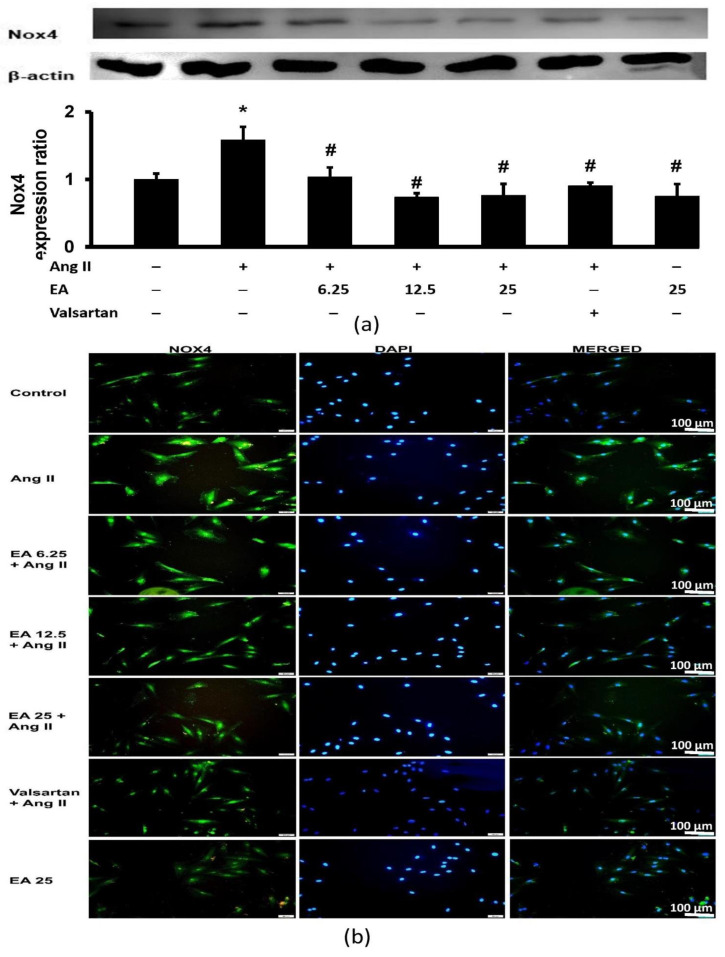
Effects of the EA fraction (µg/mL) of *P. speciosa* and valsartan (20 µM) on Ang II-induced NADPH oxidase 4 (Nox4) (**a**) protein expression and (**b**) immunofluorescent expression (green), while 4′,6-diamidino-2-phenylindole (DAPI) staining in blue indicates the nuclei in H9c2 cardiomyocytes. Data are expressed as means ± SEM (*n* = 3). * *p* < 0.05 compared to control, ^#^ *p* < 0.05 compared to Ang II.

Ang II significantly increased Nox4 expression in H9c2 cardiomyocytes. The effects were inhibited by the ethyl acetate fraction and valsartan (Figure 7a). A similar observation was made with Nox4 immunofluorescence (Figure 7b). Nox4 expression was detected in the membrane, cytoskeleton, and nucleus. Induction by Ang II significantly intensified the fluorescence of Nox4 (green), while treatment with the ethyl acetate fraction decreased fluorescence.

### 3.7. Effect of Ethyl Acetate Fraction on Ang II-Induced MAPK Signaling Pathway Activation

**Figure 8:**
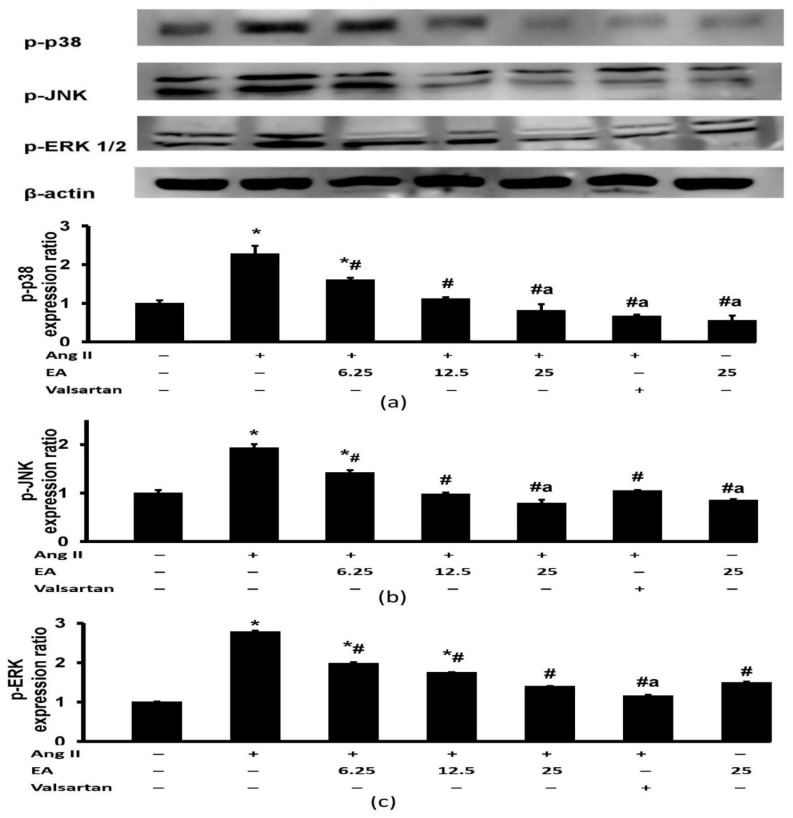
Effects of the EA fraction (µg/mL) of *P. speciosa* and valsartan (20 µM) on the Ang II-induced MAPK signaling pathway, namely (**a**) p-p38, (**b**) p-ERK 1/2, and (**c**) p-JNK, in H9c2 cardiomyocytes. Data are expressed as means ± SEM (*n* = 3). * *p* < 0.05 compared to control, ^#^ *p* < 0.05 compared to Ang II, ^a^ *p* < 0.05 compared to EA at 6.25 µg/mL + Ang II.

Ang II induced a marked increase in the expression of p-p38, p-JNK, and p-ERK1/2 (Figure 8). Treatment with the ethyl acetate fraction attenuated the increased p-p38 and p-JNK induced by Ang II in a concentration-dependent manner (Figure 8a,b). The expression of p-ERK1/2 was similarly attenuated in the groups treated with different concentrations of ethyl acetate fraction (Figure 8c). The group treated with valsartan exhibited significant protective effects on p-p38, p-JNK, and p-ERK1/2 expression but had a remarkably lower p-p38 and p-ERK1/2 expression than the group treated with the lowest concentration of ethyl acetate fraction (6.25 µg/mL).

### 3.8. Effect of Ethyl Acetate Fraction on Ang II-Induced Calcineurin Related Pathway Activation

**Figure 9:**
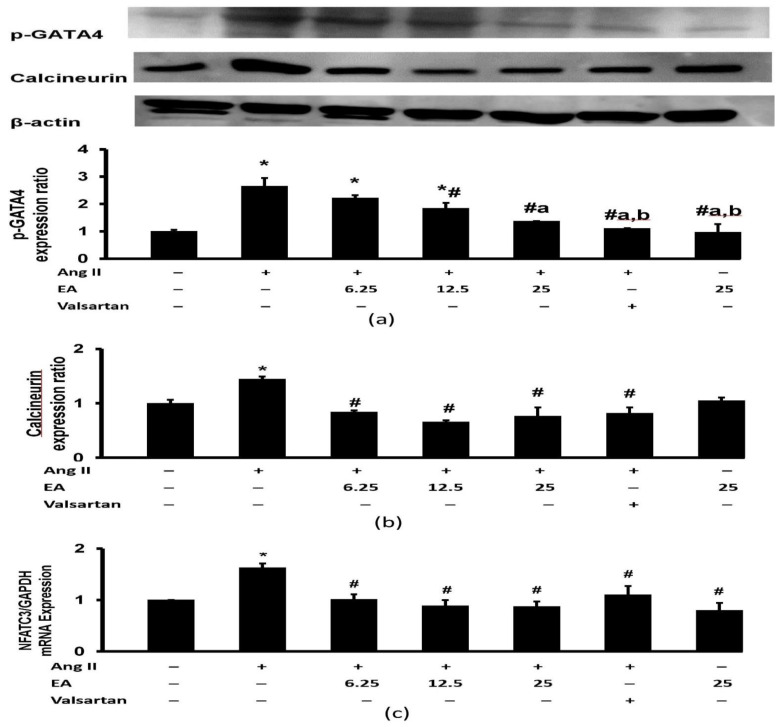
Effects of the EA fraction (µg/mL) of *P. speciosa* and valsartan (20 µM) on (**a**) p-GATA4 and (**b**) calcineurin protein expression and (**c**) NFATC3 gene expression in Ang II-induced cardiomyocyte hypertrophy. Data are expressed as means ± SEM (*n* = 3). * *p* < 0.05 compared to control, ^#^ *p* < 0.05 compared to Ang II, ^a^ *p* < 0.05 compared to 6.25 + Ang II, ^b^ *p* < 0.05 compared to 12.5 + Ang II.

Ang II increased protein expression of p-GATA4 in H9c2 cells (Figure 9a). Treatment with ethyl acetate fraction reduced the detrimental effect of Ang II on p-GATA4 in a concentration-dependent manner. Furthermore, valsartan inhibited Ang II-induced p-GATA expression, and the expression was remarkably lower than in the groups treated with the 6.25 µg/mL and 12.5 µg/mL ethyl acetate fraction.

Ang II significantly increased calcineurin protein expression, which was reduced by treatment with ethyl acetate fraction and valsartan (Figure 9b). No difference was noted between treatment groups. A similar finding was observed with NFATC3 gene expression, where Ang II increased expression, and the effect was inhibited by the ethyl acetate fraction and valsartan (Figure 9c).

## 4. Discussion

In the current study, crude ethanol extract of *P. speciosa* empty pods and its ethyl acetate and methanol fractions exhibited antihypertrophic properties in H9c2 cardiomyocytes exposed to Ang II. The ethyl acetate fraction exhibited the most intense effect. The findings suggest that the bioactive constituents responsible for the effect were semipolar and polar compounds, which could be phenolics, flavonoids, and terpenoids, which were putatively identified by UHPLC-QTOF-MS/MS. In a previous study, a different set of metabolites was identified using a similar chromatography method in crude ethanol extracts of *P. speciosa* empty pods collected from the same site [23]. Theaflavin, myricitrin, curcumin, and tremulacin were additionally identified. Previous studies have reported the antioxidant and anti-inflammatory properties of ethyl acetate fraction of the empty pods in human umbilical vein endothelial cells and cardiomyocytes [18,19,20]. Quercetin was also detected in the fraction [18,20]. Quercetin 3′-glucoside and quercetin 3-(6‴-sinapylglucosyl)(1->2)-galactoside were putatively identified in the ethyl acetate fraction of the present study. Oxidative stress and inflammation play a prominent role in the development of myocardial hypertrophy [29]. However, the cardioprotective effect of the ethyl acetate fraction was not seen at the highest concentration (200 µg/mL). It was possible that the concentration might have posed a toxic effect, manifested in the increase in cell size. Moreover, the number of viable cells at the concentration was reduced to approximately 80%. On the other hand, the cardioprotective effects of the methanol fraction at higher concentrations were investigated at 100–300 µg/mL. It also demonstrated protective effects against Ang II on cell surface area, total protein content, and intracellular ROS level (Figures S1–S3), which were comparable to those of ethyl acetate fraction.

The protective effect of the ethyl acetate fraction against Ang II-induced cardiomyocyte hypertrophy was evidenced by decreased cell surface area, protein content, and ANP expression. These are typical features of cardiac hypertrophy [27,30]. The increase in protein content occurs due to increased myocardial RNA content, leading to cardiac hypertrophy in animals exposed to pressure overload [31]. Hypertrophy activates multiple fetal cardiac genes, including ANP and BNP, causing their release in response to augmented volume-mediated stretch of the myocardial cells [32]. The fraction exerted better inhibitory effect than valsartan on ANP expression, proposing the protective mechanism was partially different from that of valsartan, likely not via Ang II type 1 receptor blockade. ANP-converting enzyme (corin) activates inactive pro-ANP into ANP in the heart, while zymogen corin is activated by proprotein convertase subtilisin/kexin type 6 (PCSK6) [33]. The expression of PCSK6 was reported to be elevated in experimental myocardial infarction-induced cardiac remodeling [34]. Therefore, it is possible that the fraction decreased the activity of PCSK6, resulting in the reduced activity of corin and finally the downregulation of ANP expression. However, the postulation needs to be investigated and confirmed in future studies.

The findings of the current study suggest that the ethyl acetate fraction of *P. speciosa* empty pods effectively inhibits cardiomyocyte hypertrophic responses, and this effect was similarly reported with the crude ethanol extract [14]. The antihypertrophic activity of the crude extract was believed to be mediated by the presence of rutin and quercetin, which were detected by HPLC [14]. However, rutin was not one of the tentatively identified compounds in the ethyl acetate fraction in our study, possibly due to the difference in chromatography methods used in our study. Rutin decreased Ang II-induced cardiomyocyte hypertrophy to a similar extent as quercetin, but at a lower concentration (50 µM versus 331 µM, respectively) [35].

ROS serves as a second messenger and is perceived as a crucial trigger for cardiac hypertrophy, which eventually progresses to heart failure [36]. ROS is produced via Nox activation upon Ang II binding to Ang II type 1 receptor. Nox4 is an important mediator in the pathogenesis of heart disease. The isoform is the principal generator of mitochondrial ROS, which mediates cardiac dysfunction [37]. In the present study, the ethyl acetate fraction significantly mitigated Ang II-induced Nox4 expression and decreased the generation of intracellular ROS in cardiomyocytes, likely via the antioxidant activity of the fraction, which was reported by Gui et al. [18]. Valsartan likewise exerted antioxidant effects, as previously reported [14].

Accumulating data have suggested that the MAPK cascade is activated by ROS in cardiomyocyte hypertrophy [38,39]. The MAPK signaling cascade is initiated by the activation of G protein-coupled receptor kinases, leading to phosphorylation of multiple intracellular targets and transcription factors by the downstream members of MAPK family, namely p38, JNK, and ERK1/2 [14]. The downregulation of phosphorylated p38, JNK, and ERK expression by the ethyl acetate fraction indicated its ability to attenuate Ang II-induced activation of the MAPK signaling cascade, leading to a reduction in cardiomyocyte hypertrophy. We postulate that the fraction diminished ROS generation via its antioxidant action, leading to the inhibition of the MAPK pathway. Activated MAPK signaling implies increased inflammation [40], suggestive of anti-inflammatory property of the compounds present in the fraction. A similar inhibition of the MAPK signaling pathway by *P. speciosa* empty pod crude extract was recently reported [14].

**Figure 10:**
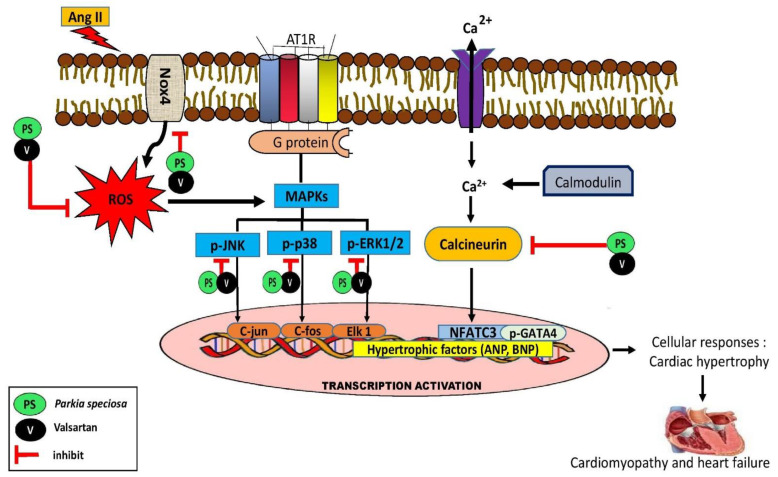
Graphical summary of potential cardioprotective effects of ethyl acetate fraction of *P. speciosa* empty pod extract against key proteins involved in cardiac hypertrophy. Ang II, Angiotensin II; AT1R, angiotensin II type 1 receptor; ANP, A-type natriuretic peptide; BNP, B-type natriuretic peptide; NFAT, nuclear factor of activated T-cells; Nox4, NADPH oxidase 4; MAPKs, Mitogen-activated protein kinases; p-ERK1/2, phosphorylated extracellular signal-related kinase; p-JNK, phosphorylated c-Jun N-terminal kinase; p-p38, phosphorylated p38 kinase; ROS, reactive oxygen species.

Upon the activation of the hypertrophic response, calcineurin activated by the presence of elevated cytosolic calcium concentration promotes dephosphorylation of p-NFATC3 and other transcription factors. NFATC3 forms a complex with GATA4, which then activates downstream transcription factors and fetal genes, such as BNP and ANP [39,41]. Treatment with the ethyl acetate fraction inhibited the increase in the expression of calcineurin, NFATC3, and p-GATA4 induced by Ang II, indicating that its cardioprotective activity may occur in part via a calcineurin-related pathway. There are no reports regarding the effects of *P. speciosa* empty pod extract on calcineurin. Physiologically, calcineurin participates in various cellular processes, including immunomodulation and cardiomyocyte maturation. However, pathologically, the activation of NFAT by calcineurin induces the activation of T cells and tumor necrosis factor α in addition to hypertrophic gene transcription, leading to pathological hypertrophy and initiation of the inflammatory process in cardiomyocytes [41]. Therefore, it can be hypothesized that the fraction inhibited the development of the inflammatory process and subsequently inhibited hypertrophic events. Moreover, GATA4 is one of the myocardial hypertrophic growth regulators [39]. Therefore, the inhibitory effect of the ethyl acetate fraction on GATA4 activation confirmed its antihypertrophic activity. Figure 10 summarizes the protective effects of ethyl acetate fraction co-treatment against cardiac hypertrophy via MAPK and calcineurin-NFAT-GATA4 signaling pathway activation. Therefore, the findings of the current study provide a basis for future studies that may identify new therapeutic targets in preventing cardiac hypertrophy.

In general, the ethyl acetate fraction did not exhibit significant concentration-dependent effects, except for the effect on ANP expression. The beneficial effects of the fraction were comparable to those of valsartan, a drug that has been used clinically to reduce cardiac remodeling and its progression to heart failure [42]. Numerous studies have demonstrated the antihypertrophic properties of some of the compounds that might be present in the fraction, such as gallic acid [43], quercetin [14], epigallocatechin-3-gallate [44], kaempferol [45], luteolin [46], and herbacetin [47].

A limitation of the current study was that the phytochemical profile of the fraction was only presumptive. However, it is believed that the protective effects observed in the study might be attributable to the presence of various bioactive compounds in the fraction. The compounds might exert synergistic effects.

## 5. Conclusions

The ethyl acetate fraction of *P. speciosa* empty pods attenuated Ang II-induced cardiomyocyte hypertrophy via its antioxidant and anti-inflammatory properties by suppressing Nox-4-associated ROS production and downregulating MAPK and calcineurin-NFAT signaling pathways. The protective effects might be attributable to the presence of flavonoids in the empty pods.

## Figures and Tables

**Table 1:** Metabolites identified from ethyl acetate fraction of *P. speciosa* empty pods by ultra-high-performance liquid chromatography-quadrupole time-of-flight mass spectrometry (UPLC-QTOF-MS/MS).

No	RT (min)	[M-H]− (m/z)	m/z	Formula	MS/MS	Tentative Identification	Class of Compound
1	1.1	169.0135	170.0208	C_7_ H_6_ O_5_	125.0278	Gallic acid	Phenolic
2	2.108	218.1041	219.1114	C_9_ H_17_ N O_5_	146.0824	Pantothenic acid	Amino acid
3	2.879	761.1266	762.1431	C_37_ H_30_ O_18_	423.0714, 591.1116	2′-2′-Bisepigallocatechin monogallate	Flavonoid
4	3.709	913.1353	914.1547	C_44_ H_34_ O_22_	177.0194, 285.0393, 423.0708, 591.1129, 743.1223	Theasinensin A	Flavonoid
5	4.132	342.1058	343.1041	C_14_ H_18_ O_7_	223.061	Picein	Phenolic
6	4.585	914.1601	915.1514	C_42_ H_34_ O_21_	169.0148, 457.0770, 703.0976	Thonningianin A	Tannin
7	7.096	463.086	464.0932	C_43_ H_32_ O_20_	300.026	3,5,7,2′,6′-Pentahydroxyflavone 2′-glucoside	Flavonoid glycoside
8	7.579	319.0465	320.0538	C_15_ H_12_ O_8_	167.0354	Amaronol A	Flavanoid
9	7.9777	463.0893	464.0963	C_21_ H_20_ O_12_	151.0032, 300.0278, 343.0461	Quercetin 3′-glucoside	Flavonoid glycoside
10	8.023	831.1714	832.2088	C_38_ H_40_ O_21_	300.0268, 463.0878	Quercetin 3-(6‴-sinapylglucosyl)(1->2)-galactoside	Flavonoid glycoside
11	8.155	463.0887	464.0958	C_21_ H_20_ O_12_	300.0276	Spiraeoside	Flavonoid glycoside
12	8.29 8.866	447.0935	448.1008	C_21_ H_20_ O_11_	301.0344 301.0346	Kaempferol 3-alpha-D-galactoside	Flavonoid glycoside
13	8.341	433.0779	434.0851	C_20_ H_18_ O_11_	151.0031, 300.0276	6-Hydroxyluteolin 7-apioside	Flavonoid glycoside
14	8.794	317.0305	318.0378	C_15_ H_10_ O_8_	178.9982	Gossypetin	Flavonoid
15	9.933	301.0365	302.0437	C_15_ H_10_ O_7_	121.0295, 151.0033, 178.9984, 229.0497	Herbacetin	Flavonoid
16	10.365	301.0359	302.0431	C_15_ H_10_ O_7_	151.0032	Bracteatin	Flavonoid
17	14.735	732.2692	733.3104	C_40_ H_47_ N O_12_	160.0144	3′-N-Debenzoyl-2′-deoxytaxol	Terpenoid
18	17.39	297.1499	298.1569	C_19_ H_22_ O_3_	183.0101	Glepidotin C	Flavonoid
19	24.398	531.4415	532.4486	C_34_ H_60_ O_4_	346.9493, 487.4492	Mayolene-16	Fatty acid

## Data Availability

The data presented in this study are available on request from the corresponding author.

## Supplementary Materials

The following supporting information can be downloaded at: https://www.mdpi.com/article/10.3390/life13010043/s1, Figure S1: Effects of the ethyl acetate (EA) and methanol (MeOH) fractions (µg/mL) of *P. speciosa* and valsartan (20 µM) on cell surface area in Ang II-induced cardiomyocyte hypertrophy.; Figure S2: Effects of the ethyl acetate (EA) and methanol (MeOH) fractions (µg/mL) of *P. speciosa* and valsartan (20 µM) on protein content per cell (mg/million cells) in Ang II-induced cardiomyocyte hypertrophy; Figure S3: Effects of the ethyl acetate (EA) and methanol (MeOH) fractions (µg/mL) of *P. speciosa* and valsartan (20 µM) on reactive oxygen species (ROS) in Ang II-induced cardiomyocyte hypertrophy.
