# Supplementary material for: Parkia speciosa Hassk. Empty Pod Extract Prevents Cardiomyocyte Hypertrophy by Inhibiting MAPK and Calcineurin-NFATC3 Signaling Pathways
Source: Life (Basel). 2022 Dec 23;13(1):43. doi: 10.3390/life13010043 (PMC9864749; doi:10.3390/life13010043)
Supplement: Supplementary file 1 [file life-13-00043-s001.zip › life-2019395-supplementary.pdf]

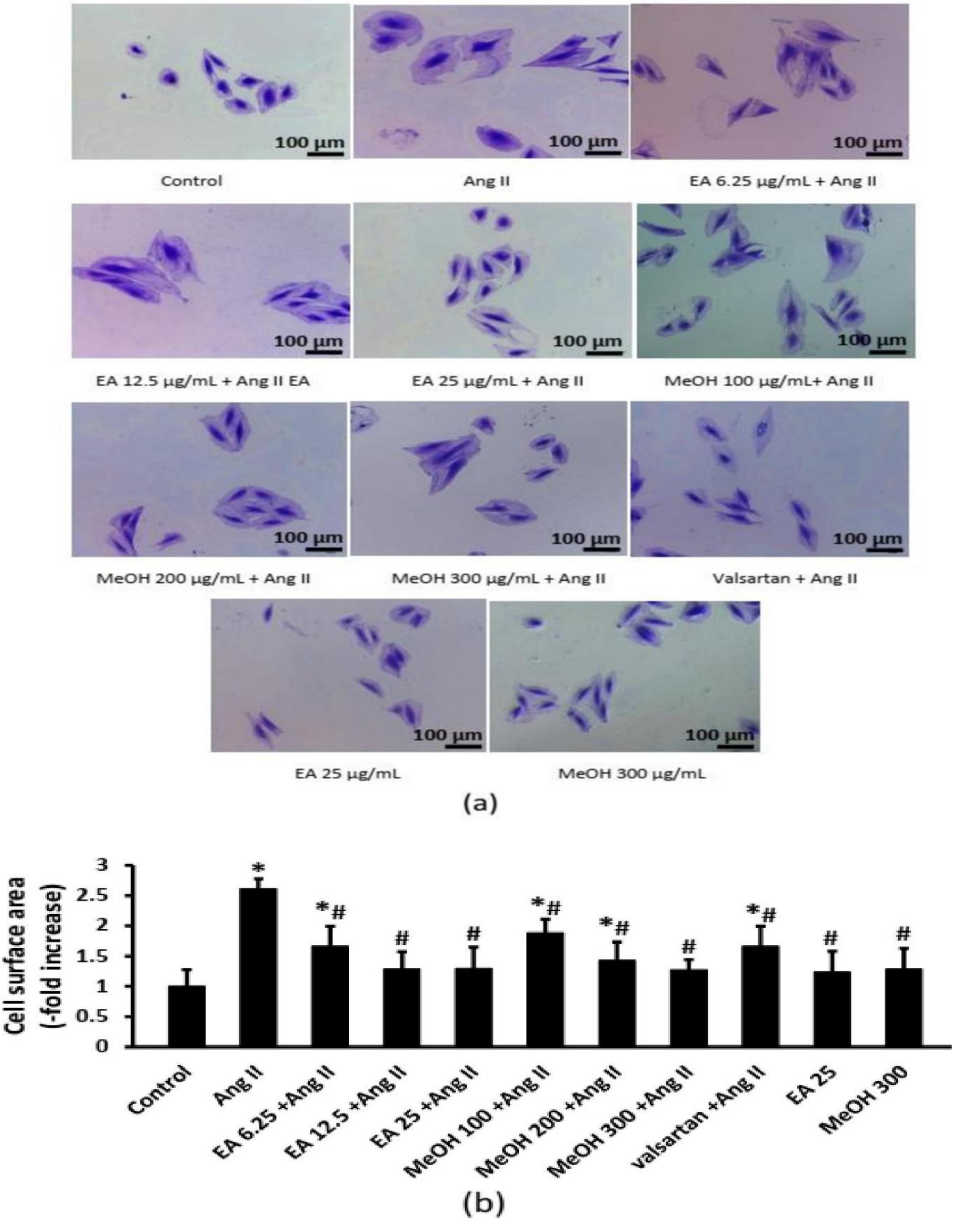

Figure S1. Effects of the ethyl acetate (EA) and methanol (MeOH) fractions (µg/mL) of *P. speciosa* and valsartan (20 µM) on cell surface area in Ang II-induced cardiomyocyte hypertrophy. (**a**) Representative images from each group at 100× magnification and (**b**) relative cell surface area. Data are expressed as means ± SEM (n = 3). * $p < 0.05$ compared to control, # $p < 0.05$ compared to Ang II.

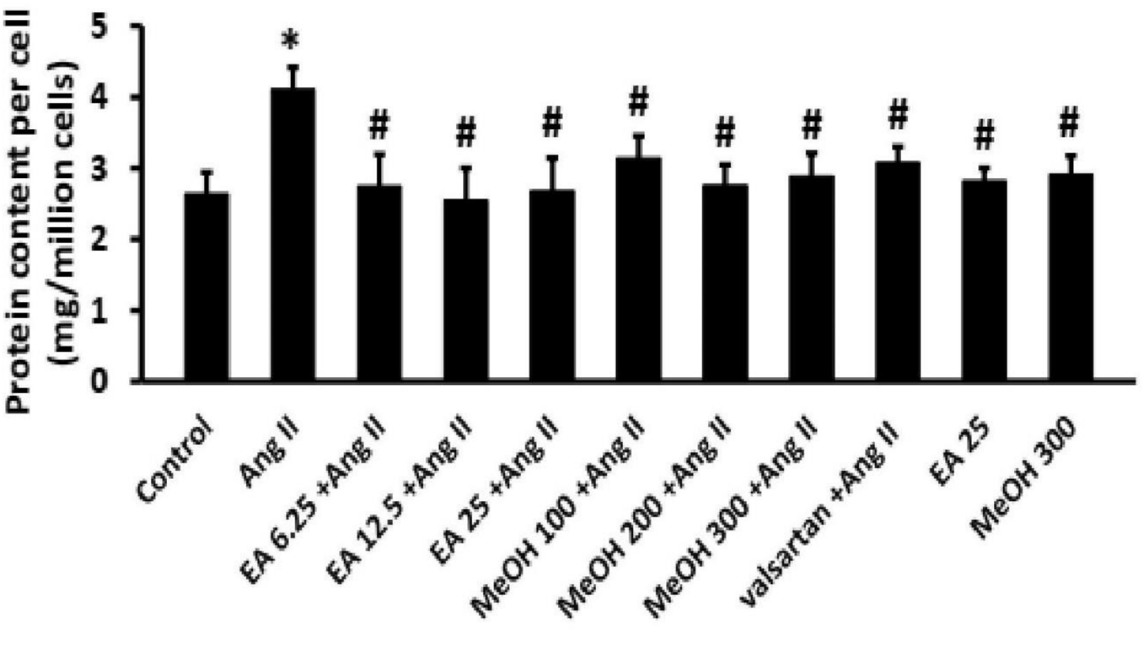

Figure S2. Effects of the ethyl acetate (EA) and methanol (MeOH) fractions (μg/mL) of *P. speciosa* and valsartan (20 μM) on protein content per cell (mg/million cells) in Ang II-induced cardiomyocyte hypertrophy. Data are expressed as means ± SEM (n = 3). * $p < 0.05$ compared to control, # $p < 0.05$ compared to Ang II.

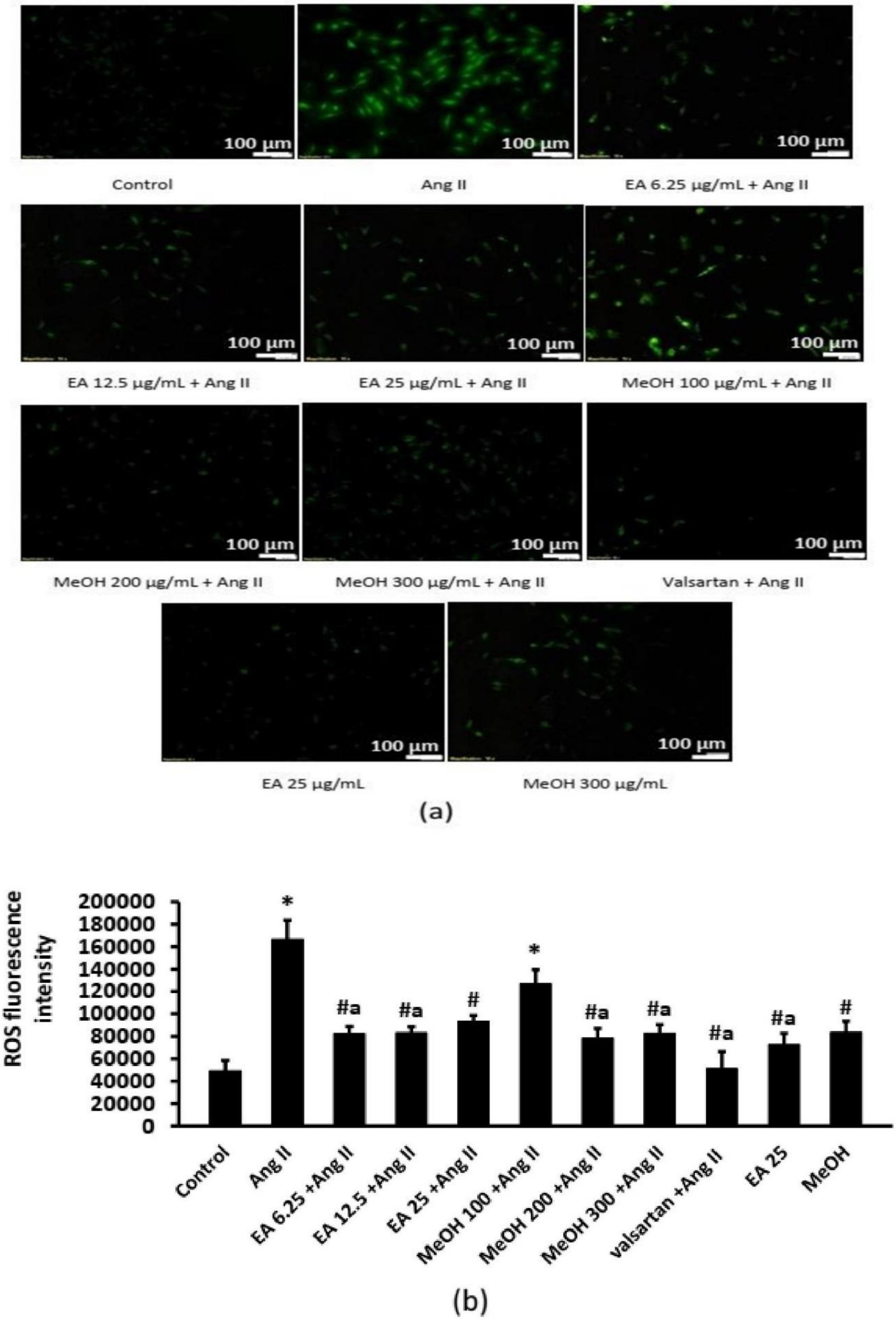

Figure S3. Effects of the ethyl acetate (EA) and methanol (MeOH) fractions (µg/mL) of *P. speciosa* and valsartan (20 µM) on reactive oxygen species (ROS) in Ang II-induced cardiomyocyte hypertrophy. (**a**) Representative images from each treatment group and (**b**) ROS fluorescence intensity. Data are expressed as means ± SEM (n = 3). * $p < 0.05$ compared to control, # $p < 0.05$ compared to Ang II, a $p < 0.05$ compared to MeOH 100 + Ang II.
